# Generation of KCL034 clinical grade human embryonic stem cell line

**DOI:** 10.1016/j.scr.2015.12.034

**Published:** 2016-01

**Authors:** Liani Devito, Laureen Jacquet, Anastasia Petrova, Cristian Miere, Victoria Wood, Neli Kadeva, Glenda Cornwell, Stefano Codognotto, Emma Stephenson, Dusko Ilic

**Affiliations:** Stem Cell Laboratories, Division of Women's Health, Faculty of Life Sciences and Medicine, King's College London and Assisted Conception Unit, Guys' Hospital, London, United Kingdom

## Abstract

The KCL034 human embryonic stem cell line was derived from a normal healthy blastocyst donated for research. The ICM was isolated using laser microsurgery and plated on γ-irradiated human foreskin fibroblasts. Both the derivation and cell line propagation were performed in an animal product-free environment and under current Good Manufacturing Practice (cGMP) standards. Pluripotent state and differentiation potential were confirmed by in vitro assays. The line was also validated for sterility, specific and non-specific human pathogens.

## 1 Resource table

**Table:** 

Name of stem cell line	KCL034
Institution	King's College London, London UK
Derivation team	Neli Kadeva, Victoria Wood, Glenda Cornwell, Stefano Codognotto, Emma Stephenson
Contact person and email	Dusko Ilic, email: dusko.ilic@kcl.ac.uk
Date archived/stock date	Aug. 08, 2011
Type of resource	Biological reagent: cell line
Sub-type	Human pluripotent stem cell line
Origin	Human embryo
Key marker expression	Pluripotent stem cell markers: NANOG, OCT4, TRA-1-60, TRA-1-81, alkaline phosphatase (AP) activity
Authentication	Identity and purity of line confirmed
Link to related literature (direct URL links and full references)	1) Jacquet, L., Stephenson, E., Collins, R., Patel, H., Trussler, J., Al-Bedaery, R., Renwick, P., Ogilvie, C., Vaughan, R., Ilic, D., 2013. Strategy for the creation of clinical grade hESC line banks that HLA-match a target population. EMBO Mol. Med. 5 (1), 10–17. doi: 10.1002/emmm.201201973 http://www.ncbi.nlm.nih.gov/pubmed/23161805
2) Canham, A., Van Deusen, A., Brison, D.R., De Sousa, P., Downie, J., Devito, L., Hewitt, Z.A., Ilic, D., Kimber, S.J., Moore, H.D., Murray, H., Kunath, T., 2015. The molecular karyotype of 25 clinical-grade human embryonic stem cells lines. *Sci. Rep.* 5, 17258. doi: 10.1038/srep17258 http://www.ncbi.nlm.nih.gov/pubmed/26607962
3) Ilic, D., Stephenson, E., Wood, V., Jacquet, L., Stevenson, D., Petrova, A., Kadeva, N., Codognotto, S., Patel, H., Semple, M., Cornwell, G., Ogilvie, C., Braude, P., 2012. Derivation and feeder-free propagation of human embryonic stem cells under xeno-free conditions. Cytotherapy. 14 (1), 122–128. doi: 10.3109/14653249.2011.623692 http://www.ncbi.nlm.nih.gov/pubmed/22029654
4) Stephenson, E., Jacquet, L., Miere, C., Wood, V., Kadeva, N., Cornwell, G., Codognotto, S., Dajani, Y., Braude, P., Ilic, D., 2012. Derivation and propagation of human embryonic stem cell lines from frozen embryos in an animal product-free environment. Nat. Protoc. 7 (7), 1366–1381. doi: 10.1038/nprot.2012.080 http://www.ncbi.nlm.nih.gov/pubmed/22722371
5) Devito, L., Petrova, A., Miere, C., Codognottom S., Blakely, N., Lovatt, A., Ogilvie, C., Khalaf, Y., Ilic, D., 2014. Cost-effective master cell bank validation of multiple clinical-grade human pluripotent stem cell lines from a single donor. Stem Cells Transl. Med. 3(10), 1116–1124. doi: 10.5966/sctm.2014–0015 http://www.ncbi.nlm.nih.gov/pubmed/25122690
6) Devito, L., Petrova, A., Miere, C., Codognottom S., Blakely, N., Lovatt, A., Ogilvie, C., Khalaf, Y., Ilic, D., 2014. Cost-effective master cell bank validation of multiple clinical-grade human pluripotent stem cell lines from a single donor. Stem Cells Transl. Med. 3(10), 1116–1124. doi: 10.5966/sctm.2014–0015 http://www.ncbi.nlm.nih.gov/pubmed/25122690
7) Petrova, A., Celli, A., Jacquet, L., Dafou, D., Crumrine, D., Hupe, M., Arno, M., Hobbs, C., Cvoro, A., Karagiannis, P., Devito, L., Sun, R., Adame, L.C., Vaughan, R., McGrath, J.A., Mauro, T.M., Ilic, D., 2014. 3D In vitro model of a functional epidermal permeability barrier from human embryonic stem cells and induced pluripotent stem cells. Stem Cell Reports. 2(5), 675–689. doi: 10.1016/j.stemcr.2014.03.009 http://www.ncbi.nlm.nih.gov/pubmed/24936454
8) Cvoro, A., Devito, L., Milton, F.A., Noli, L., Zhang, A., Filippi, C., Sakai, K., Suh, J.H., Sieglaff, D., Dhawan, A., Sakai, T., Ilic, D., Webb, P., 2015. A thyroid hormone receptor/KLF9 axis in human hepatocytes and pluripotent stem cells. Stem Cells. 33(2), 416–428. doi: 10.1002/stem.1875 http://www.ncbi.nlm.nih.gov/pubmed/25330987
Information in public databases	KCL034 is a National Institutes of Health (NIH) registered hESC line
NIH Registration Number: NIHhESC-14-0268
http://grants.nih.gov/stem_cells/registry/current.htm?id=654
Ethics	The hESC line KCL034 is derived under licence from the UK Human Fertilisation and Embryology Authority (research licence numbers: R0075 and R0133) and also has local ethical approval (UK National Health Service Research Ethics Committee Reference: 06/Q0702/90).
Informed consent was obtained from all subjects and the experiments conformed to the principles set out in the WMA Declaration of Helsinki and the NIH Belmont Report. No financial inducements are offered for donation.

## 2 Resource details

**Table:** 

Consent signed	May 26, 2009
Embryo thawed	Jul. 11, 2011
UK Stem Cell Bank Deposit Approval	Mar. 08, 2012 Reference: SCSC12-54
Sex	Male 46, XY
Grade	Clinical
Disease status	Healthy/unaffected
Karyotype (aCGH)	No copy number changes detected.
SNP Array	Gain in region 6p22.1 (Canham et al., 2015)
DNA fingerprint	Allele sizes (in bp) of 16 microsatellite markers specific for chromosomes 13, 18 and 21 (Jacquet et al., 2013)
HLA typing	HLA-A 11,29; B 44,51; Bw 4; C 04,16; DRB1 04,07; DRB4 01; DQB1 02,03 (Jacquet et al., 2013, Canham et al., 2015)
Viability testing	Pass
Mycoplasma	Negative
Sterility	Pass
Pluripotent markers (immunostaining) (Fig. 1)	NANOG, OCT4, TRA-1-60, TRA-1-81, AP activity
Three germ layers differentiation in vitro (immunostaining) (Fig. 2)	Endoderm: AFP Ectoderm: TUBB3 (tubulin, beta 3 class III) Mesoderm: ACTA2 (actin, alpha 2, smooth muscle)
Three germ layer differentiation in vivo (teratomas) (Fig. 3)	Endoderm: AFP, GATA4 Ectoderm: TUBB3, GFAP (glial fibrillary acidic protein) Mesoderm: DES (desmin), Alcian Blue and periodic acid–Schiff (PAS)-stained cartilage
Targeted differentiation (Fig. 4)	Endoderm: definitive endoderm — GATA4 (Cvoro et al., 2015). Ectoderm: keratinocytes — p63, KRT14 (Petrova et al., 2014) Mesoderm: cardiomyocytes — TNNT2
Sibling lines available	KCL032, KCL033

**Fig. 1:**
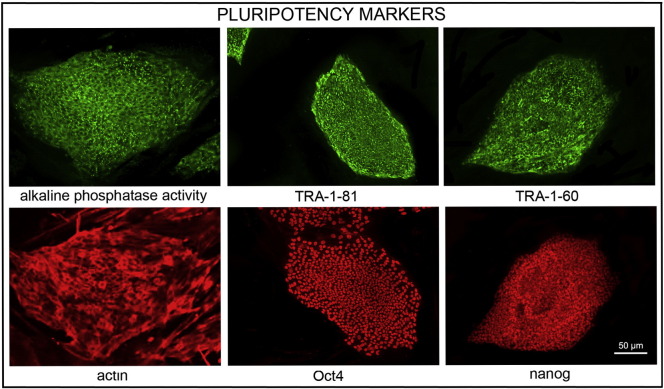
Expression of pluripotency markers. Pluripotency is confirmed by immunostaining (Oct4, Nanog, TRA-1-60, TRA-1-81) and alkaline phosphatase (AP) activity assay. Actin stress fibers, visualized with rhodamine–phalloidin (red), are present in both feeders and hES cell colonies, whereas AP activity (green) is detected only in hES cells. Scale bar, 50 μm.

**Fig. 2:**
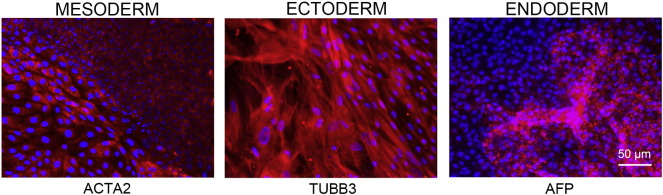
Differentiation of three germ layers in vitro is confirmed by detection of markers: smooth muscle actin (ACTA2, red) for mesoderm, β-III tubulin (TUBB3, red) for ectoderm and α-fetoprotein (AFP, red) for endoderm. Nuclei are visualized with Hoechst 33342 (blue). Scale bar, 50 μm.

**Fig. 3:**
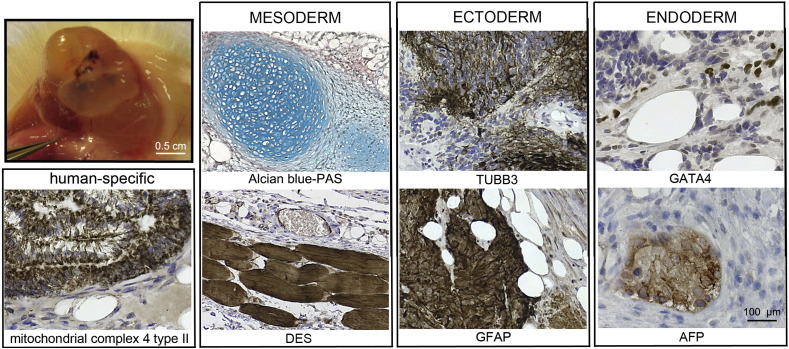
Differentiation of three germ layers in vivo. Teratomas were encapsulated and did not invade surrounding tissue. Sections are counterstained with hematoxylin and eosin and specific stains are brown (immunohistochemistry) or light blue (Alcian blue). Germ layer markers: Alcian blue–PAS-stained cartilage and DES for mesoderm, TUBB3 and GFAP for ectoderm, GATA4 and AFP for endoderm. Positive immunostaining for complex IV type II marker confirms the human origin of the tumor (adjacent section of the one stained for desmin). Scale bars are 100 μm.

**Fig. 4:**
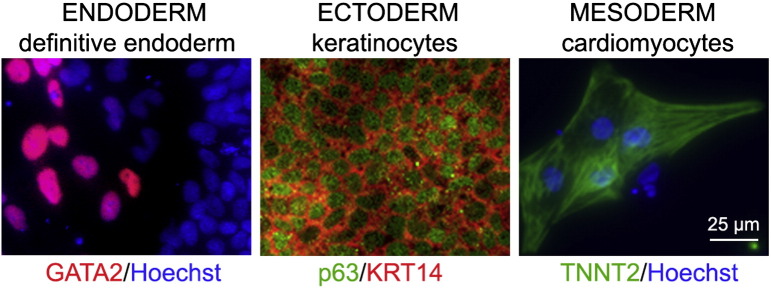
Targeted differentiation into specific cell types of endoderm (definitive endoderm), extoderm (keratinocytes) and mesoderm (cardiomyocytes). Definitive endoderm: GATA2 (red), nuclei (blue). Ectoderm: p63 (red), KRT14 (green), nuclei (blue). Mesoderm: TNNT2 (green), nuclei (blue). Scale bar, 125 μm.

We generated KCL034 clinical grade hESC line following protocols, established previously (Ilic et al., 2012, Stephenson et al., 2012), and now adapted to cGMP conditions. The expression of the pluripotency markers was tested after freeze/thaw cycle (Fig. 1). Differentiation potential into three germ layers was verified in vitro (Fig. 2), in vivo (Fig. 3) and with targeted differentiation into specific endoderm, ectoderm and mesoderm cell types (Fig. 4).

Molecular karyotyping identified a gain on chromosome 6p22.1. The gain on chromosome 5p14.3 containing the following genes: *HIST1H2BL*, *HIST1H2AI*, *HIST1H3H*, *HIST1H2AJ*, *HIST1H2BM*, *HIST1H4J*, *HIST1H4K*, *HIST1H2AK*, *HIST1H2BN*, *HIST1H2AL*, *HIST1H1B*, *HIST1H3I*, *HIST1H4L*, *HIST1H3J*, *HIST1H2AM*, *HIST1H2BO*, *OR2B2* and *OR2B6* (Canham et al., 2015). The 330.8 kb gain starts at bp 27627265 and ends at bp 27958049 as referred to Human Genome Build 38. This duplication that contained part of the Histone 1 gene cluster was not fully present on the database of genomic variants (DGV; http://dgv.tcag.ca), which has collected structural variations in more than 14,000 healthy individuals from worldwide population (MacDonald et al., 2014). It is probable that this gain represents a benign event as other histone clusters have been shown to be preferentially duplicated during evolution (Canham et al., 2015, Braastad et al., 2004).

Validation for sterility and specific and non-specific human pathogens (Devito et al., 2014) conformed that the cells in Master Bank were sterile, mycoplasma-free, and negative as well as for Treponema pallidum, Chlamydia, Neisseria gonorrhoeae, Human immunodeficiency virus-1 and 2 (HIV-1 and -2), Human T-lymphotropic virus type-1 and 2 (HTLV-1 and 2), Hepatitis A, B and C (HAV, HBV and HCV), Human herpes simplex virus HHV-4 (Epstein–Barr virus, EBV), -6, -7, and -8, Human cytomegalovirus (hCMV), human parvovirus B19, SV40, JCV, BKV, Enterovirus, HAV, HCV, nonspecific viral and other adventitious contaminants.

We also generated research grade of KCL034 line that is adapted to feeder-free conditions.

## 3 Materials and methods

### 3.1 Consenting process

We distribute Patient Information Sheet (PIS) and consent form to the in vitro fertilization (IVF) patients if they opted to donate to research embryos that were stored for 5 or 10 years. They mail signed consent back to us and that might be months after the PIS and consent were mailed to them. If in meantime new versions of PIS/consent are implemented, we do not send these to the patients or ask them to re-sign; the whole process is done with the version that was given them initially. The PIS/consent documents (FRO-V.6) were created on Dec. 18, 2008. HFEA Code of Practice that was in effect at the time of document creation: Edition 7 — R.4 (http://www.hfea.gov.uk/2999.html). The donor couple signed the consent on May 26, 2009. HFEA Code of Practice that was in effect at the time of donor signature: Edition 7 — R.4. HFEA Code of Practice Edition 7 — R.4 was in effect: 02 Oct. 2008–30 Sep. 2009.

### 3.2 Embryo culture and micromanipulation

Embryo culture and laser-assisted dissection of inner cell mass (ICM) were carried out as previously described in details (Ilic et al., 2012, Stephenson et al., 2012). The cellular area containing the ICM was then washed and transferred to plates containing mitotically inactivated human neonatal foreskin fibroblasts (HFF).

### 3.3 Cell culture

ICM plated on mitotically inactivated HFF were cultured as described (Ilic et al., 2012, Stephenson et al., 2012). TE cells were removed mechanically from outgrowth (Ilic et al., 2007, Ilic et al., 2010). hES colonies were expanded and cryopreserved at the third passage.

### 3.4 Viability test

Straws with the earliest frozen passage (p. 2–3) are thawed and new colonies are counted three days later. These colonies are then expanded up to passage 8, at which point cells were part frozen and part subjected to standard battery of tests (pluripotency markers, in vitro and in vivo differentiation capability, genetics, sterility, mycoplasma).

### 3.5 Pluripotency markers

Pluripotency was assessed using two different techniques: enzymatic activity assay [alkaline phosphatase (AP) assay] and immunostaining as described (Ilic et al., 2012, Stephenson et al., 2012).

### 3.6 Differentiation

Spontaneous differentiation into three germ layers was assessed in vitro and in vivo as described (Petrova et al., 2014, Stephenson et al., 2012). Targeted differentiation in cardiomyocytes (Jacquet et al., 2015, Laflamme et al., 2007) and definitive endoderm (Cvoro et al., 2015, Cheng et al., 2012), keratinocytes (Petrova et al., 2014), followed the protocols described earlier. Nuclei are visualized with Hoechst 33342.

### 3.7 Genotyping

DNA was extracted from hESC cultures using a Chemagen DNA extraction robot according to the manufacturer's instructions. Amplification of polymorphic microsatellite markers was carried out as described (Ilic et al., 2012). Allele sizes were recorded to give a unique fingerprint of each cell line.

### 3.8 Array comparative genomic hybridization (aCGH)

aCGH was performed as described in details (Ilic et al., 2012).

### 3.9 Whole-genome single nucleotide polymorphism (SNP) array

SNP array was performed as described in details (Canham et al., 2015).

### 3.10 HLA typing

HLA-A, -B and -DRB1 typing was performed with a PCR sequence-specific oligonucleotide probe (SSOP; Luminex, Austin, TX, USA) hybridization protocol at the certified Clinical Transplantation Laboratory, Guy's and St Thomas' NHS Foundation Trust and Serco Plc. (GSTS) Pathology (Guy's Hospital, London, UK) as described (Jacquet et al., 2013). HLA typing was also performed independently by other group (Canham et al., 2015).

### 3.11 Validation for sterility and specific and non-specific human pathogens

Validation for sterility and specific and non-specific human pathogens was performed as described (Devito et al., 2014). All validation studies were conducted by SGS Vitrology (Glasgow, U.K., http://www.sgs.com), in compliance with the principles of GMP as set out in Directive 2003/94/EC for medicinal products for human use (Directive 2003/94/EC, 2003) and 91/412/EEC for veterinary medicinal products (Directive 91/412/EEC, 1991).

Sterility testing was performed in accordance with the current requirements of the European Pharmacopoeia, Section 2.6.1 Sterility, U.S. Pharmacopeia, 71. Sterility Tests, and International Conference on Harmonisation Topic Q5D guidelines.

Mycoplasma testing was performed in accordance with the current requirements of the European Pharmacopoeia, Section 2.6.7, Mycoplasmas.

All PCR-based assays used were compliant with the current edition of the European Pharmacopoeia, 2.6.21, Nucleic Acid Amplification Techniques.

## 4 Author disclosure statement

There are no competing financial interests in this study.
